# Multicenter evaluation of a single-device dengue antibody/antigen rapid test for simultaneous detection of NS1, IgM, and IgG

**DOI:** 10.1128/spectrum.03669-25

**Published:** 2026-05-18

**Authors:** Zhihai Zhao, Mettingal Ramakrishnan Shincy, Lay Sin Teoh, Kadahalli Lingegowda Ravikumar, Haris Ong, Matthew Mak, Yang Du, Mohammad Shafiul Alam, Na Xu

**Affiliations:** 1 Department of Research and Development, MP Biomedicals Asia Pacific Pte Ltd, Singapore, Singapore; 2 Central Research Laboratory, Kempegowda Institute of Medical Sciences 29135, Bangalore, India; 3 Infectious Diseases Division, International Centre for Diarrhoeal Diseases Research, Bangladesh (icddr, b) 56291, Dhaka, Bangladesh; National Chung Hsing University, Taichung, Taiwan (Province of China)

**Keywords:** dengue, rapid diagnostic test, dengue NS1, dengue IgG/IgM, dengue serology, ASSURE Dengue Ab/Ag Rapid Test

## Abstract

Dengue is a major global health burden in tropical and subtropical regions, where timely diagnosis is crucial for clinical management and outbreak control. Although rapid diagnostic tests (RDTs) are widely used at the point of care, their performance varies considerably. This study evaluated the diagnostic accuracy of the ASSURE Dengue Ab/Ag Rapid Test across three independent settings: Singapore, India, and Bangladesh, using reference enzyme-linked immunosorbent assays (ELISAs) and Conformité Européenne (CE)-marked RDTs. In Singapore, 430 characterized serum/plasma samples (141 positive and 289 negative) were tested against Panbio ELISAs and compared with the SD Standard Q Dengue Duo. In India, 300 prospective samples were tested against Panbio ELISAs. In Bangladesh, 85 retrospective samples were evaluated against commercial RDTs, ELISAs, and PCR. In Singapore, ASSURE achieved 100% sensitivity and specificity, outperforming SD Standard Q (sensitivity, 46.81%; specificity, 99.31%). In India, perfect concordance with Panbio ELISAs was observed across non-structural protein 1 (NS1), immunoglobulin M (IgM), and immunoglobulin G (IgG). In Bangladesh, NS1 detection was consistently strong (sensitivity, 100%; specificity, 88.14%–96.00%, depending on the comparator), while antibody detection showed lower sensitivity (IgM, 45.45%; IgG, 42.50%) but high specificity (91.07%–100%). Overall, the ASSURE Dengue Ab/Ag Rapid Test demonstrated superior diagnostic performance compared with CE-marked ELISAs and RDTs, particularly for NS1 antigen detection, and maintained accuracy across multicenter evaluations. These findings support its use as a reliable point-of-care tool for early dengue diagnosis.

**IMPORTANCE:**

Dengue fever is a mosquito-borne viral infection that affects millions annually. Early diagnosis is critical to reduce complications and improve patient outcomes. While rapid diagnostic tests (RDTs) are widely used, their reliability remains inconsistent. We evaluated the ASSURE Dengue Ab/Ag Rapid Test in Singapore, India, and Bangladesh, comparing it to established laboratory-based enzyme-linked immunosorbent assays and other commercial RDTs. Across studies, the test consistently achieved high accuracy, particularly for non-structural protein 1 antigen detection, which is crucial for identifying acute dengue infection. In contrast, the antibody components (immunoglobulin M and immunoglobulin G) showed variable sensitivity, highlighting the well-documented weaknesses and challenges that dengue tests currently face, particularly in antibody detection. Taken together, these results demonstrate that ASSURE is a dependable point-of-care test, especially for early-phase dengue diagnosis, and can improve patient management and outbreak response in endemic regions.

## INTRODUCTION

Dengue fever, caused by the dengue virus (DENV) and transmitted primarily by *Aedes* mosquitoes, represents a major global public health challenge (1). With an estimated 390 million infections annually, of which approximately 96 million manifest clinically, dengue is endemic in over 100 countries across tropical and subtropical regions, including parts of Asia, the Americas, Africa, and the Pacific (2). The World Health Organization classifies dengue as a neglected tropical disease, with outbreaks escalating in frequency and severity due to factors such as urbanization, climate change, and international travel (3). Past epidemics, such as those in Jamaica (2012), Malaysia (2015), and Reunion Island (2018–2019), have highlighted the circulation of dengue virus strains with epidemic potential or increased virulence, with case fatality rates reaching up to 0.28% in severe outbreaks (4–7).

Dengue infection presents a spectrum of clinical manifestations, ranging from asymptomatic or mild flu-like symptoms to severe forms like dengue hemorrhagic fever and dengue shock syndrome, which can be fatal without timely intervention (8). Diagnostic markers are useful for identifying the stage of infection, but individual markers reflect different aspects. The non-structural protein 1 (NS1) is a highly conserved glycoprotein secreted by infected cells and is a reliable marker of acute viremia. It is detectable in serum at an early stage of infection (often from the first day of fever) and up to about 9–10 days and is therefore useful for acute-phase diagnosis rather than for monitoring disease progression (9). Immunoglobulin M (IgM) antibodies typically appear by days 3–5 post-onset, peaking around days 10–14 and persisting for 2–3 months, indicating recent infection (10). Immunoglobulin G (IgG) responses arise later in primary infection (around days 7–10) but typically rise rapidly and to higher levels during secondary infections and then remain detectable for years (10, 11). Combining these markers, NS1 for early viremia and IgM/IgG for serological response, enhances diagnostic coverage across infection stages.

Despite advances in diagnostics, dengue detection cases face significant challenges, particularly in resource-limited settings during outbreaks. Molecular methods like reverse transcription polymerase chain reaction offer high sensitivity (up to 95%) and specificity for viral RNA but require specialized equipment, trained personnel, and several hours to days for results, rendering them impractical for point-of-care use (12). Enzyme-linked immunosorbent assays (ELISAs) for NS1, IgM, or IgG are more accessible and accurate (sensitivities often >80%) but are similarly time-consuming (2–4 hours), costly, and laboratory-dependent (13). Rapid diagnostic tests (RDTs), designed for quick results (15–30 minutes) without instrumentation, address these gaps but often exhibit inconsistent reliability, with sensitivities varying widely (e.g., 42%–90% for NS1 and 38%–90% for IgM) due to factors like serotype differences, infection stage, and cross-reactivity with other flaviviruses such as Zika (ZIKV) or Chikungunya (14–18).

The novel ASSURE Dengue Ab/Ag Rapid Test aims to overcome these limitations by providing a combined detection of IgG, IgM, and NS1 antigen with high accuracy, as validated against the robust Panbio Dengue ELISA standard, which is widely recognized for its reliability in serological and antigen detection (15, 17, 19). The ASSURE Dengue Ab/Ag Rapid Test was therefore further subjected to a clinical validation in India to independently validate its consistency with the Panbio Dengue ELISA standard. To investigate its performance against other commercial dengue tests, comparison studies among other commercial dengue tests were conducted in Bangladesh, where SD Bioline Dengue Duo, DENV Detect IgM Capture ELISA (InBios International, Inc.), VIASURE Zika, Dengue & Chikungunya Real Time PCR Detection Kit, and OnSite Dengue Ag Rapid Test (CTK Biotech) were available.

## MATERIALS AND METHODS

### Study design

The diagnostic performance of the ASSURE Dengue Ab/Ag Rapid Test (MP Biomedicals Asia Pacific Pte Ltd, Singapore), a lateral flow immunochromatographic assay for the qualitative detection of dengue NS1 antigen, IgM antibodies, and IgG antibodies in human serum, plasma, or whole blood, was evaluated in three independent studies: (i) an in-house study at MP Biomedicals Asia Pacific Pte Ltd (Singapore); (ii) an external study at the Central Research Laboratory, Kempegowda Institute of Medical Sciences, Bangalore, India; and (iii) an external study at the International Centre for Diarrhoeal Disease Research, Bangladesh (icddr,b).

The in-house study aimed to characterize dengue samples using reference ELISAs and determine the performance of the ASSURE test, including comparison with a Conformité Européenne (CE)-marked dengue rapid test, the SD Biosensor Standard Q Dengue Duo, and assessment of interference and potential cross-reactivity in special samples. The Indian study was a prospective cross-sectional evaluation of the ASSURE test against reference ELISAs, with comparison to respective Panbio ELISAs. The Bangladesh study was a retrospective evaluation using samples with known dengue status, comparing the ASSURE test to various RDTs, ELISAs, and PCR.

### Clinical samples

In the in-house study, a total of 141 dengue-positive and 289 dengue-negative single serum samples were used. Positive samples were from patients with confirmed dengue infection, while negative samples were from healthy donors or patients with other conditions. All samples were characterized using reference ELISAs (described below). Additionally, to assess potential interference, 39 dengue-negative samples were tested, including 5 antinuclear antibody, 4 bilirubin, 4 triglyceride, 5 icteric, 4 total protein, 4 lipemic, 4 hemolyzed, and 9 rheumatoid factor-positive samples sourced from BiosPacific and SLR. For the analytical sensitivity study, the negative plasma used as diluent was from Access Biologicals. The live dengue virus culture samples (DENV-1 to DENV-4) were from HeavyBio.

In the Indian study, 300 serum samples were collected from patients with suspected dengue and either tested fresh or stored at −80°C. The panel included 100 NS1-positive and 100 NS1-negative samples characterized by Panbio Dengue Early ELISA; 75 IgG-positive and 100 IgG-negative samples characterized by Panbio Dengue IgG Indirect ELISA; and 25 IgM-positive and 100 IgM-negative samples characterized by Panbio Dengue IgM Capture ELISA. The 100 negative samples were the same set used for all marker evaluations, confirmed negative for NS1, IgM, and IgG.

In the Bangladesh study, 85 serum samples were used. The number of samples varied by comparator test (85 for OnSite Dengue Ag Rapid Test; 75 for Bioline Dengue Duo NS1 Ag; 67 for DENV Detect IgM Capture ELISA and Bioline Dengue Duo IgG/IgM; and 50 for VIASURE Zika, Dengue & Chikungunya Real-Time PCR Detection Kit) (20).

### Reference tests and comparators

In the in-house study, samples were characterized using three CE-marked ELISAs: Panbio Dengue IgG Capture ELISA (catalog 01PE10, lot 01P10H005), Panbio Dengue IgM Capture ELISA (catalog 01PE20, lot 01P20I006), and Panbio Dengue Early ELISA for NS1 (catalog 01PE40, lot 01P4H014) (all from Abbott Diagnostics Korea Inc.). Primary dengue was defined as NS1 and/or IgM positive with undetectable IgG, whereas secondary dengue was defined as NS1 and/or IgM positive with detectable IgG. The ASSURE test was compared to the CE-marked commercial RDT, SD Biosensor Standard Q Dengue Duo (SD Biosensor).

In the Indian study, reference tests were Panbio Dengue Early ELISA for NS1, Panbio Dengue IgG Indirect ELISA for IgG, and Panbio Dengue IgM Capture ELISA for IgM (Abbott).

In the Bangladesh study, comparators for NS1 included OnSite Dengue Ag Rapid Test (CTK Biotech), Bioline Dengue Duo NS1 Ag (Abbott), and VIASURE Zika, Dengue & Chikungunya Real Time PCR Detection Kit (Certest Biotec); comparators for IgM included Bioline Dengue Duo IgG/IgM (Abbott) and DENV Detect IgM Capture ELISA (InBios International, Inc.); and the comparator for IgG was Bioline Dengue Duo IgG/IgM (Abbott).

All reference RDTs, ELISAs, and PCR were performed by trained personnel in virology laboratories following manufacturers’ instructions for use (IFU).

### Test procedures

**Fig 1:**
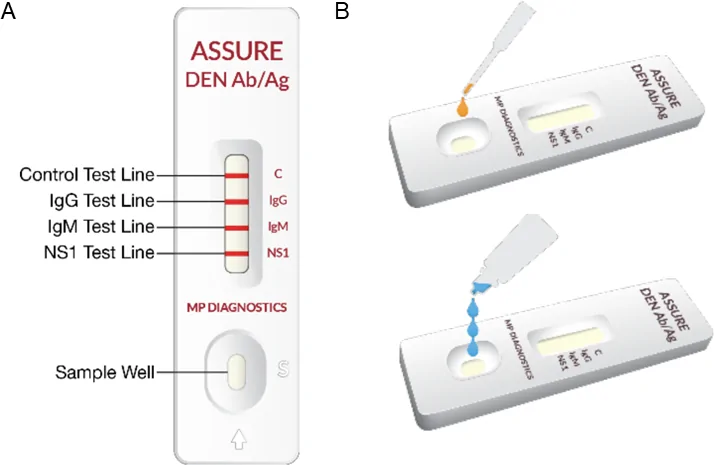
ASSURE Dengue Ab/Ag Rapid Test device layout and test procedures. (**A**) The test strip detects NS1 antigen, IgM, and IgG antibodies. Each test line indicates the presence of the corresponding marker, with a control line ensuring assay validity. (**B**) Schematics of test procedures. Briefly, 30 μL of serum/plasma/whole blood sample is added to the sample well, followed by three drops of assay buffer, and results are read at 15–20 minutes. A colored control line validated the test; a colored test line indicated positive for NS1, IgM, or IgG, respectively. To ensure accurate and consistent interpretation of test results, a standardized reading procedure was implemented. Tests were primarily interpreted by a single trained reader. For any test where a line appeared faint or its presence was ambiguous, two additional trained readers, independently and blinded to the initial interpretation and reference results, evaluated the test. The final interpretation for these cases was determined by majority agreement among the three readers. This process was established to minimize subjectivity in classifying samples with weak or borderline reactivity.

The ASSURE Dengue Ab/Ag Rapid Test was performed according to the manufacturer’s instructions (Fig. 1). Comparator RDTs were used according to their respective IFU. All tests were conducted by trained personnel, and results were interpreted visually without knowledge of reference results to avoid bias.

### Statistical analysis

Diagnostic performance was evaluated by calculating sensitivity, specificity, positive predictive value (PPV), negative predictive value (NPV), and accuracy, with 95% confidence intervals (CIs) using MedCalc (MedCalc Software Ltd., Ostend, Belgium). The analyses presented are descriptive in nature, focusing on performance metrics and categorical agreement; no inferential statistical tests were performed that would require adjustment for multiple comparisons.

## RESULTS

As summarized in Table 1, a total of 430 samples (141 dengue-positive and 289 dengue-negative) were characterized using three reference ELISAs (Panbio Dengue IgG Capture, IgM Capture, and Early ELISA for NS1) in the primary testing site. The 141 positive samples were classified into 41 primary infections (negative for IgG and positive for IgM and/or NS1) and 100 secondary infections (positive for IgG).

**TABLE 1:** Characterization of dengue-positive samples using Panbio ELISA kits

Infection status	Country of origin	Sample size (*n*)	Panbio Dengue IgG Capture ELISA	Panbio Dengue IgM Capture ELISA	Panbio Dengue Early ELISA
Primary infection	Philippines	11	0	11	0
	Indonesia	2	0	2	0
	Bangladesh	28	0	20	17
	Total	41	0	33	17
Secondary infection	Philippines	55	55	51	0
	Indonesia	1	1	1	0
	Singapore	1	1	0	0
	Bangladesh	41	41	36	4
	Malaysia	2	2	2	0
	Total	100	100	90	4

The ASSURE Dengue Ab/Ag Rapid Test demonstrated perfect diagnostic performance against the Panbio ELISA reference standard, showing 100% sensitivity (141/141; 95% CI: 97.42%–100%) and 100% specificity (289/289; 95% CI: 98.73%–100%), resulting in an overall accuracy of 100% (Table 2).

**TABLE 2:** Diagnostic performance of the ASSURE Dengue Ab/Ag Rapid Test compared to the Panbio ELISA reference standard (Singapore study)

Diagnostic performance	ASSURE Dengue Ab/Ag Rapid Test	
	Value	95% CI
Sensitivity (*n* = 141)	100% (141/141)	97.42%–100%
Specificity (*n* = 289)	100% (289/289)	98.73%–100%
PPV	100%	97.42%–100%
NPV	100%	98.73%–100%
Accuracy	100%	99.15%–100%

As shown in Table 3, the ASSURE Dengue Ab/Ag Rapid Test maintained 100% specificity across all challenging negative sample cohorts, including healthy donors (*n* = 117), hospitalized patients (*n* = 41), pregnant individuals (*n* = 36), and crucially, panels containing potential cross-reactive agents (hepatitis A/C, measles IgG, chikungunya, malaria, yellow fever, Zika, *Helicobacter pylori*, and tuberculosis; *n* = 56) and common clinical interference substances (antinuclear antibody, bilirubin, triglyceride, icteric, total protein, lipemic, hemolyzed, and rheumatoid factor; *n* = 39). The total number of samples across all categories sums to 289.

**TABLE 3:** Specificity performance of ASSURE Dengue Ab/Ag Rapid Test across challenging negative sample cohorts (Singapore study)

Sample category	Sample profile	Sample size (*n*)	ASSURE Dengue Ab/Ag Rapid Test specificity (95% CI)
General populations	Healthy donors	117	100% (117/117), (96.76%–100.00%)
	Hospitalized patients	41	100% (41/41), (91.40%–100.00%)
	Pregnancy	36	100% (36/36), (90.26%–100.00%)
Cross-reactive samples	Hepatitis A	10	100% (10/10)
	Hepatitis C	6	100% (6/6)
	Measles IgG	9	100% (9/9)
	Chikungunya	10	100% (10/10)
	Malaria	7	100% (7/7)
	Yellow fever	4	100% (4/4)
	Zika	2	100% (2/2)
	*H. pylori*	4	100% (4/4)
	Tuberculosis	4	100% (4/4)
	Total cross-reactive	56	100% (56/56), (93.64%–100.00%)
Interference substances	Antinuclear antibody	5	100% (5/5)
	Bilirubin	4	100% (4/4)
	Triglyceride	4	100% (4/4)
	Icteric	5	100% (5/5)
	Total protein	4	100% (4/4)
	Lipemic	4	100% (4/4)
	Hemolyzed	4	100% (4/4)
	Rheumatoid factor	9	100% (9/9)
	Total interference	39	100% (39/39) (91.01%–100.00%)
Overall total		289	100% (289/289) (98.73%–100.00%)

A comparative evaluation with CE-marked commercial RDT, SD Biosensor Standard Q Dengue Duo, was conducted. The ASSURE test significantly outperformed the competitor when compared to the Panbio ELISA. Its overall accuracy of 100% surpassed that of the SD Standard Q (82.09%) tests (Table 4). The ASSURE test’s sensitivity (100%) was markedly higher than SD Standard Q (46.81%) (Table 4).

**TABLE 4:** Comparative diagnostic performance of the ASSURE Dengue Ab/Ag Rapid Test and the SD Standard Q Dengue Duo test (Singapore study)

Test kit	Sensitivity (*n* = 141)	Specificity (*n* = 289)	Accuracy
ASSURE Dengue Ab/Ag	100% (141/141)	100% (289/289)	100% (430/430)
	97.42%–100%	98.73%–100%	99.15%–100%
SD Standard Q Dengue Duo	46.81% (66/141)	99.31% (287/289)	82.09% (353/430)
	(38.36%–55.39%)	(97.52%–99.92%)	(78.14%–85.60%)

The comparative performance of the ASSURE Dengue Ab/Ag Rapid Test and SD Standard Q Dengue Duo Rapid Test, evaluated against the ELISA reference standard for detecting primary and secondary dengue infections, revealed significant differences in accuracy. As shown in Table 5, for primary dengue infection (*n* = 41), the ASSURE Dengue test achieved a high accuracy of 95.1% (39/41), detecting 39 samples with visible test lines across various combinations (2 IgG + IgM, 9 IgM + NS1, 22 IgM ONLY, and 8 NS1 ONLY), closely aligning with the ELISA reference (9 IgM + NS1, 24 IgM ONLY, and 8 NS1 ONLY). In contrast, the SD Standard Q test showed a lower accuracy of 68.3% (28/41), detecting 28 samples (4 IgM + NS1, 11 IgM ONLY, and 13 NS1 ONLY) but missing 13 samples (no visible lines).

**TABLE 5:** Band profile analysis between ASSURE Dengue Ab/Ag Rapid Test and SD Standard Q Dengue Duo Rapid Test in the primary and secondary infection groups (Singapore study)

Metric	ELISA (reference)	ASSURE Dengue Ab/Ag Rapid Test	SD Standard Q Dengue Duo Rapid Test
Primary dengue infection (*n* = 41)			
No visible line	0	0	13
IgG ONLY	0	0	0
IgG + IgM	0	2	0
IgG + NS1	0	0	0
IgG + IgM + NS1	0	0	0
IgM + NS1	9	9	4
IgM ONLY	24	22	11
NS1 ONLY	8	8	13
Total detected	41	39	28
Accuracy (%)		95.12 (39/41)	68.29 (28/41)
(95% CI)		(83.47%–99.40%)	(51.91%–81.92%)
Secondary dengue infection (*n* = 100)			
No visible line	0	0	62
IgG ONLY	10	10	1
IgG + IgM	86	85	12
IgG + NS1	0	0	1
IgG + IgM + NS1	4	5	1
IgM + NS1	0	0	0
IgM ONLY	0	0	21
NS1 ONLY	0	0	2
Total detected	100	100	15
Accuracy (%)		100 (100/100)	15.00 (15/100)
(95% CI)		(96.38%–100.0%)	(8.65%–23.53%)

For secondary dengue infection (*n* = 100), the ASSURE Dengue test demonstrated perfect accuracy of 100% (100/100), detecting all 100 samples (10 IgG ONLY, 85 IgG + IgM, and 5 IgG + IgM + NS1), matching the ELISA reference (10 IgG ONLY, 86 IgG + IgM, and 4 IgG + IgM + NS1). The SD Standard Q test, however, had a significantly lower accuracy of 15% (15/100), detecting only 15 samples (1 IgG ONLY, 12 IgG + IgM, 1 IgG + NS1, 1 IgG + IgM + NS1, 21 IgM ONLY, and 2 NS1 ONLY) and failing to detect 85 samples (21 IgM ONLY and 2 NS1 ONLY, no visible lines). Overall, these findings highlight the superior performance of the ASSURE Dengue test over the SD Standard Q test in accurately detecting both primary and secondary dengue infections.

In an independent external evaluation conducted in India, the ASSURE Dengue Ab/Ag Rapid Test demonstrated perfect concordance with the reference Panbio ELISAs across all analytes, fully aligning with the findings from the in-house study. As summarized in Table 6, the test achieved 100% sensitivity, specificity, PPV, NPV, and overall accuracy for NS1, IgM, and IgG detection, with narrow confidence intervals supporting the robustness of the results. Specifically, for NS1, both sensitivity and specificity were 100% (95% CI: 96.38%–100.00%); for IgM, sensitivity was 100% (95% CI: 86.28%–100.00%) and specificity was 100% (95% CI: 96.38%–100.00%); and for IgG, sensitivity was 100% (95% CI: 95.20%–100.00%) and specificity was 100% (95% CI: 96.38%–100.00%). Collectively, these findings from the Indian study reinforce and validate the in-house findings, demonstrating that the ASSURE Dengue Ab/Ag Rapid Test provides consistently high diagnostic accuracy across independent study settings.

**TABLE 6:** Comparative diagnostic performance of the ASSURE Dengue Ab/Ag Rapid Test (NS1, IgM, and IgG) versus Panbio ELISA assays in an independent Indian study

Test analyte	Sample size (*n*)	Sensitivity (95% CI)	Specificity (95% CI)	PPV (95% CI)	NPV (95% CI)	Accuracy (95% CI)
NS1	100 positives + 100 negatives	100.00% (96.38%–100.00%)	100.00% (96.38%–100.00%)	100.00% (96.38%–100.00%)	100.00% (96.38%–100.00%)	100.00% (98.17%–100.00%)
IgM	25 positives + 100 negatives	100.00% (86.28%–100.00%)	100.00% (96.38%–100.00%)	100.00% (86.28%–100.00%)	100.00% (96.38%–100.00%)	100.00% (97.09%–100.00%)
IgG	75 positives + 100 negatives	100.00% (95.20%–100.00%)	100.00% (96.38%–100.00%)	100.00% (95.20%–100.00%)	100.00% (96.38%–100.00%)	100.00% (97.91%–100.00%)

In a third independent evaluation conducted in Bangladesh, the ASSURE Dengue Ab/Ag Rapid Test continued to demonstrate highly reliable NS1 antigen detection, confirming its consistent diagnostic performance across diverse settings. As shown in Table 7, against the OnSite Dengue Ag Rapid Test, sensitivity was 100.00% (95% CI: 86.77%–100.00%) with a specificity of 88.14% (95% CI: 77.07%–95.09%). Compared to the molecular reference VIASURE PCR, the test maintained 100.00% sensitivity (95% CI: 86.28%–100.00%) and a high specificity of 96.00% (95% CI: 79.65%–99.90%). Against the Bioline Dengue NS1 Ag test, sensitivity remained 100.00% (95% CI: 86.28%–100.00%), with a specificity of 96.00% (95% CI: 79.65%–99.09%). Across all comparisons, NPV consistently reached 100%, highlighting the test’s ability to accurately identify dengue-negative cases. These findings, alongside the in-house Singapore and external Indian studies, demonstrate that the ASSURE Dengue Ab/Ag Rapid Test provides consistently strong NS1 detection performance across multiple independent sites, reinforcing its reliability and suitability as a diagnostic tool in varied endemic contexts.

**TABLE 7:** Performance of the dengue NS1 antigen test line of MP Diagnostics ASSURE Dengue Rapid Test in the Bangladesh Study against the respective dengue antigen tests

Reference test	Sample size (*n*)	Sensitivity (95% CI)	Specificity (95% CI)	PPV (95% CI)	NPV (95% CI)	Accuracy (95% CI)
OnSite Dengue Ag Rapid Test	26 positives + 59 negatives	100.00% (86.77%–100.00%)	88.14% (77.07%–95.09%)	78.79% (64.95%–88.16%)	100.00% (93.15%–100.00%)	91.76% (83.77%–96.62%)
VIASURE PCR	25 positives + 25 negatives	100.00% (86.28%–100.00%)	96.00% (79.65%–99.90%)	96.15% (78.56%–99.42%)	100.00% (85.75%–100.00%)	98.00% (89.35%–99.95%)
Bioline Dengue NS1 Ag	25 positives + 50 negatives	100.00% (86.28%–100.00%)	96.00% (86.29%–99.51%)	92.59% (76.27%–97.98%)	100.00% (92.60%–100.00%)	97.33% (90.70%–99.68%)

The IgM performance of the ASSURE (MP) Dengue Test and the Bioline Dengue IgG/IgM test line was also evaluated in the Bangladesh study (*n* = 67) using the DENV Detect IgM Capture ELISA from Inbios as the reference standard (Table 8). The ASSURE test achieved an overall accuracy of 83.58% (56/67; 95% CI: 72.52%–91.51%), which was notably higher than the Bioline test at 73.13% (49/67; 95% CI: 60.90%–83.24%). While the Bioline assay demonstrated perfect sensitivity of 100% (11/11; 95% CI: 71.51%–100.0%), this was offset by its poor specificity of 67.86% (38/56; 95% CI: 54.04%–79.71%), resulting in a low PPV of only 37.93% (11/29; 95% CI: 29.46%–47.2%). In contrast, the ASSURE test showed balanced performance with a sensitivity of 45.45% (5/11; 95% CI: 16.75%–76.62%) and a higher specificity of 91.07% (51/56; 95% CI: 80.38%–97.04%). The ASSURE test’s moderate IgM sensitivity may reflect reported specificity limitations of the reference DENV Detect IgM Capture ELISA; this is examined further in Discussion. This highlights the importance of reference test characteristics when evaluating new diagnostics. Table S1 lists the ASSURE test band profiles for samples with discrepant results compared to the reference method.

**TABLE 8:** Comparative IgM detection performance of the ASSURE and SD Bioline Dengue tests against Inbios DENV Detect IgM Capture ELISA (Bangladesh study)

Test	Sample size (*n*)	Sensitivity (95% CI)	Specificity (95% CI)	PPV (95% CI)	NPV (95% CI)	Accuracy (95% CI)
ASSURE Dengue Ab/Ag Rapid Test	11 positives + 56 negatives	45.45% (16.75%–76.62%)	91.07% (80.38%–97.04%)	50.00% (25.77%–74.23%)	89.47% (83.12%–93.62%)	83.58% (72.52%–91.51%)
SD Bioline Dengue Duo		100.00% (71.51%–100.00%)	67.86% (54.04%–79.71%)	37.93% (29.46%–47.20%)	100.00% (90.75%–100.00%)	73.13% (60.90%–83.24%)

In the Bangladesh-based study (Table 9), the IgG performance of the ASSURE Dengue Ab/Ag Rapid Test was compared with the SD Bioline Dengue IgG/IgM test line. Among 67 evaluable samples, the ASSURE test detected 17 true IgG positives that were also identified by SD Bioline, but it classified an additional 23 SD Bioline positives as negative. This yielded an overall sensitivity of 42.5% (17/40) and a specificity of 100% (27/27). The PPV was therefore 100%, while the NPV was 54%. Importantly, ASSURE produced no false positives, and all IgG positives it detected were concordant with SD Bioline. This pattern is consistent with the test’s design, where the IgG reactivity was calibrated to specifically detect the higher antibody titers characteristic of secondary infections, mirroring the threshold of the Panbio Dengue IgG Capture ELISA. The band profiles for discrepant results from the ASSURE test are provided in Table S2.

**TABLE 9:** Comparative IgG detection performance of the ASSURE Dengue Ab/Ag Rapid Test versus the SD Bioline Dengue Duo IgG test line (Bangladesh study)

Contingency table		SD Bioline Dengue Duo IgG test line		
		Positive	Negative	Total
ASSURE Dengue Ab/Ag Rapid Test	Positive	17	0	17
	Negative	23	27	50
Total		40	27	67
Performance metrics			Value	95% CI
Sensitivity			42.50%	27.04%–59.11%
Specificity			100%	87.23%–100.00%
PPV			100%	80.49%–100.00%
NPV			54%	47.35%–60.51%

To further investigate the performance of ASSURE Dengue Ab/Ag Rapid Test, additional analytical sensitivity studies were performed using live dengue virus culture samples representing all four serotypes (DENV-1 to DENV-4), which were quantified in focus-forming units per milliliter (ffu/mL). ASSURE demonstrated superior analytical sensitivity across all dengue virus serotypes (Table 10). The NS1 limit of detection for ASSURE was 2.5 × 10³ ffu/mL for DENV-1, DENV-3, and DENV-4, and 5 × 10³ ffu/mL for DENV-2 in dengue-negative plasma. In contrast, SD Bioline Dengue Duo and SD Biosensor Standard Q Dengue Duo generally required higher viral concentrations. Collectively, these controlled dilution experiments using live virus-derived NS1 antigen provide objective, quantitative evidence that the ASSURE Dengue Ab/Ag Rapid Test exhibits superior analytical sensitivity, independent of clinical classification.

**TABLE 10:** Analytical sensitivity comparison for dengue NS1 antigen detection using serial dilutions of live virus culture (DENV-1 to DENV-4) in plasma tested with ASSURE, SD Bioline, and Standard Q rapid tests

Dengue virus serotype	Dilution factor (concentration, ffu/mL)	Replicate	ASSURE Dengue Ab/Ag Rapid Test^*a*^				SD Bioline Dengue Duo (NS1 device)^*a*^		SD Biosensor Standard Q Dengue Duo (NS1 device)^*a*^	
			Control	IgG	IgM	NS1	Control	NS1	Control	NS1
DENV-1	Neat (2 × 10^4^)	1	Present	−	−	++	Present	+	Present	+
		2	Present	−	−	++	Present	+	Present	+
	1:2 (1 × 10^4^)	1	Present	−	−	+	Present	+	Present	+
		2	Present	−	−	+	Present	+	Present	+
	1:4 (5 × 10^3^)	1	Present	−	−	+	Present	−	Present	−
		2	Present	−	−	+	Present	−	Present	−
	1:8 (2.5 × 10^3^)	1	Present	−	−	+	Present	−	Present	−
		2	Present	−	−	+	Present	−	Present	−
	1:16 (1.25 × 10^3^)	1	Present	−	−	+	NT	NT	NT	NT
		2	Present	−	−	−	NT	NT	NT	NT
	1:32 (6.25 × 10^2^)	1	Present	−	−	−	NT	NT	NT	NT
		2	Present	−	−	−	NT	NT	NT	NT
DENV-2	Neat (2 × 10^4^)	1	Present	−	−	+	Present	+	Present	+
		2	Present	−	−	+	Present	+	Present	+
	1:2 (1 × 10^4^)	1	Present	−	−	+	Present	−	Present	+
		2	Present	−	−	+	Present	−	Present	+
	1:4 (5 × 10^3^)	1	Present	−	−	+	Present	−	Present	+
		2	Present	−	−	+	Present	−	Present	+
	1:8 (2.5 × 10^3^)	1	Present	−	−	−	NT	NT	Present	−
		2	Present	−	−	+	NT	NT	Present	−
	1:16 (1.25 × 10^3^)	1	Present	−	−	−	NT	NT	Present	−
		2	Present	−	−	−	NT	NT	Present	−
	1:32 (6.25 × 10^2^)	1	Present	−	−	−	NT	NT	NT	NT
		2	Present	−	−	−	NT	NT	NT	NT
DENV-3	Neat (2 × 10^4^)	1	Present	−	−	++	Present	+	Present	++
		2	Present	−	−	++	Present	+	Present	++
	1:2 (1 × 10^4^)	1	Present	−	−	+	Present	+	Present	+
		2	Present	−	−	+	Present	+	Present	+
	1:4 (5 × 10^3^)	1	Present	−	−	+	Present	+	Present	+
		2	Present	−	−	+	Present	+	Present	+
	1:8 (2.5 × 10^3^)	1	Present	−	−	+	Present	−	Present	+
		2	Present	−	−	+	Present	−	Present	+
	1:16 (1.25 × 10^3^)	1	Present	−	−	+	Present	−	Present	−
		2	Present	−	−	−	Present	−	Present	−
	1:32 (6.25 × 10^2^)	1	Present	−	−	−	NT	NT	Present	−
		2	Present	−	−	−	NT	NT	Present	−
DENV-4	Neat (2 × 10^4^)	1	Present	−	−	++	Present	+	Present	+
		2	Present	−	−	++	Present	+	Present	+
	1:2 (1 × 10^4^)	1	Present	−	−	+	Present	+	Present	+
		2	Present	−	−	+	Present	+	Present	+
	1:4 (5 × 10^3^)	1	Present	−	−	+	Present	−	Present	−
		2	Present	−	−	+	Present	−	Present	−
	1:8 (2.5 × 10^3^)	1	Present	−	−	+	Present	−	Present	−
		2	Present	−	−	+	Present	−	Present	−
	1:16 (1.25 × 10^3^)	1	Present	−	−	−	NT	NT	NT	NT
		2	Present	−	−	−	NT	NT	NT	NT
	1:32 (6.25 × 10^2^)	1	Present	−	−	−	NT	NT	NT	NT
		2	Present	−	−	−	NT	NT	NT	NT

^*a*^ Symbols indicate reactivity levels: +, weak reactive; ++, moderate to strong reactive; −, negative; NT, not tested.

To confirm the test’s compatibility with sample types suitable for point-of-care use, a formal specimen equivalency study was performed. Matched sets of venous whole blood, finger-prick capillary whole blood, plasma, and serum were obtained from individual donors. The study evaluated both native negative samples and contrived positive samples, where negative matrices were spiked with characterized positive materials for dengue IgG, IgM, and NS1 antigen. Testing in duplicate demonstrated 100% concordance in final interpretation across all four specimen types (Tables S3 and S4; Fig. S1). The intensities of the specific test lines showed only minimal variation (±0.5 intensity score) between matrices. This study validates that the ASSURE Dengue Ab/Ag Rapid Test delivers equivalent performance when using finger-prick whole blood, venous whole blood, plasma, or serum.

## DISCUSSION

The combined evidence from multicenter evaluations confirms that the ASSURE Dengue Ab/Ag Rapid Test delivers superior accuracy compared with widely used CE-marked RDTs. The 100% sensitivity and specificity observed in the controlled data set highlight its robust design, while the independent third-party evaluation confirmed these findings in real-world clinical settings. Importantly, the assay performed consistently across infection stages, detecting both primary and secondary infections with high reliability, a critical advantage since secondary infections are associated with severe disease progression. Its negligible cross-reactivity and strong resistance to interference further enhance its clinical utility, making it a dependable tool for both patient management and surveillance programs.

A key benefit is the ability to simultaneously detect NS1 antigen, IgM, and IgG antibodies within a single assay. This integrated format provides clinicians with immediate information on both acute viral presence (via NS1) and the host immune response (via IgM/IgG), which can support differentiation between early acute infection/primary infection and secondary infection patterns when interpreted alongside clinical presentation. Consolidating these three markers into one device reduces the need for multiple separate tests, simplifies workflow, shortens turnaround time, and can potentially lower overall testing cost.

The NS1 component of the ASSURE Dengue Rapid Test demonstrated excellent diagnostic performance, achieving perfect sensitivity (100%) across all comparator methods, including OnSite Dengue Ag Rapid Test, SD Bioline Dengue NS1, and, most importantly, the molecular gold standard VIASURE PCR. This consistent ability to correctly identify all true NS1-positive cases underscores the robustness of the assay for detecting acute dengue infection. The slightly lower specificity against OnSite may be attributable to cross-reactivity or limitations of the OnSite test itself, rather than shortcomings of ASSURE, given that specificity improved markedly when evaluated against PCR and Bioline Dengue NS1 Ag Test. Taken together, these findings highlight the NS1 line of the ASSURE Dengue Rapid Test as a highly dependable marker for early-phase dengue diagnosis, offering clinicians a rapid and accurate tool for timely case detection and management, particularly during the critical acute stage when intervention and patient monitoring are most impactful.

Within the limitations of the multicenter studies, the performance profile of the ASSURE Dengue Ab/Ag Rapid Test must be interpreted through the critical lens of comparator choice. The stark contrast between its near-perfect agreement with the Panbio ELISA in the primary study (100% sensitivity/specificity) and its lower agreement with other tests in the supplementary study is not a contradiction but rather a reflection of the well-documented variability among commercial dengue diagnostic assays (21–23). Since ASSURE had demonstrated 100% concordance with the Panbio ELISA in both in-house and independent external evaluations, establishing Panbio as a reliable reference anchor, the apparent discordance on IgM detection between ASSURE and InBios should not be interpreted as a weakness of ASSURE. Instead, it is consistent with the established limitations of the InBios IgM kit when compared against the more sensitive Panbio ELISA (22). In other words, the results reaffirm that the ASSURE Dengue Ab/Ag Rapid Test maintains performance characteristics equivalent to Panbio, and the observed differences with InBios are in line with published data showing only fair agreement between InBios and Panbio (22). This consistency supports the robustness of ASSURE as a reliable diagnostic tool.

In the Bangladesh study, the ASSURE Dengue Ab/Ag Rapid Test demonstrated an IgG sensitivity of 42.5% and perfect specificity (100%) when compared against the Bioline Dengue IgG line, yielding a PPV of 100% and an NPV of 54%. This apparent reduction in sensitivity is not indicative of a performance deficit but is a direct result of the ASSURE test’s finely tuned IgG reactivity, which was calibrated to detect only the higher antibody titers characteristic of secondary dengue infections, mirroring the established threshold of the Panbio Dengue IgG Capture ELISA. Additionally, the ASSURE test may correctly classify samples with lower or cross-reactive IgG levels, which are flagged as negative by the less-specific Bioline assay. Independent evaluations have further documented the variable and suboptimal performance of the Bioline test in certain settings (21, 23). Therefore, the observed difference in IgG detection primarily reflects the intentionally higher specificity threshold of the ASSURE test rather than a limitation in its analytical performance.

Furthermore, a fundamental challenge for dengue serological diagnostics is the well-documented cross-reactivity among flaviviruses, driven by shared antigenic epitopes. Prior infection with related viruses such as ZIKV, West Nile virus, or yellow fever virus (YFV) can elicit antibodies that cross-react in dengue immunoassays (24–26), particularly affecting IgM and IgG detection. This established serological pattern must be considered when interpreting antibody results, especially in regions with co-circulation or historical exposure to multiple flaviviruses. In this study, preliminary testing with the limited number of available ZIKV (*n* = 2) and YFV (*n* = 4) virus-positive samples showed no cross-reactivity with the ASSURE test. Additionally, sera from individuals vaccinated against dengue and from healthy donors in non-endemic regions were not included, as such panels were unavailable. Therefore, while the current specificity results are promising, the observed performance is inherently contextual. Future evaluations incorporating larger panels from diverse flavivirus-endemic regions, post-vaccination sera, and specimens from non-endemic populations are crucial to fully characterize potential cross-reactivity and to definitively establish the test’s specificity across varied epidemiological and immunization backgrounds. Moreover, other limitations include the use of commercially sourced or historical samples in some studies, which lacked detailed clinical metadata such as infection serotype, day post-onset, or primary versus secondary infection status. These factors may affect the interpretation of dengue antigen and antibody kinetics.

While the analytical sensitivity studies using live dengue virus cultures provided valuable comparative data across all four DENV serotypes, it is important to acknowledge a key limitation in the use of focus-forming units per milliliter as a proxy for NS1 antigen levels. The ffu quantifies infectious viral particles capable of forming foci in cell culture, but the amount of NS1 antigen secreted during viral infection may vary significantly depending on the viral strain, cell line, and culture conditions used (27–30). A single focus-forming unit represents one infectious viral particle, not a standardized amount of NS1 antigen. However, the parallel testing of multiple commercial rapid diagnostic tests (SD Bioline Dengue Duo and SD Biosensor Standard Q Dengue Duo) using the same viral culture samples validated NS1 presence and provided a basis for comparing relative antigen levels across different platforms under identical testing conditions.

Last but not least, the variable performance of the antibody components is a well-recognized challenge in dengue serology, heavily influenced by the choice of comparator. The ASSURE test demonstrates high accuracy when validated against a robust standard like Panbio ELISA. Its lower agreement with other tests is primarily a function of those tests’ documented limitations in sensitivity and specificity or differences in assay design. This underscores the importance of using well-validated reference standards and interpreting RDT performance in the context of the broader diagnostic landscape.

### Conclusion

The ASSURE Dengue Ab/Ag Rapid Test demonstrated superior diagnostic accuracy across both controlled laboratory and independent clinical settings. Its robust sensitivity, specificity, and ability to classify infection stages position it as a valuable tool for dengue diagnosis in endemic regions. As a reliable point-of-care test, it enables early detection, facilitating timely clinical management, reducing mortality, and supporting outbreak containment through targeted vector control and patient isolation. In epidemic-prone regions, such tools can alleviate healthcare burdens, support surveillance, and inform public health responses, ultimately contributing to global efforts to mitigate the dengue pandemic.

## Supplementary Material

Reviewer comments

## SUPPLEMENTAL MATERIAL

Supplemental material spectrum.03669-25-s0001.pdf Tables S1 to S4; Fig. S1.

OPEN PEER REVIEW reviewer-comments.pdf An accounting of the reviewer comments and feedback.

ASM does not own the copyrights to Supplemental Material that may be linked to, or accessed through, an article. The authors have granted ASM a non-exclusive, world-wide license to publish the Supplemental Material files. Please contact the corresponding author directly for reuse.
