# Supplementary material for: Multicenter evaluation of a single-device dengue antibody/antigen rapid test for simultaneous detection of NS1, IgM, and IgG
Source: Microbiol Spectr. 2026 May 18;14(7):e03669-25. doi: 10.1128/spectrum.03669-25 (PMC13339962; doi:10.1128/spectrum.03669-25)
Supplement: Supplemental material — Tables S1 to S4; Fig. S1. [file spectrum.03669-25-s0001.pdf]

TABLE S1. Detailed ASSURE test band profiles (NS1, IgM, IgG) for samples with discrepant IgM results when compared to the DENV Detect IgM Capture ELISA reference (Bangladesh study)

Number	DENV Detect IgM Capture ELISA	ASSURE Dengue Ab/Ag Rapid Test		
		NS1	IgM	IgG
#1	Negative	Positive	Positive	Negative
#2	Negative	Negative	Positive	Negative
#3	Negative	Positive	Positive	Negative
#4	Negative	Negative	Positive	Positive
#5	Negative	Negative	Positive	Positive
#6	Positive	Negative	Negative	Positive
#7	Positive	Negative	Negative	Positive
#8	Positive	Negative	Negative	Positive
#9	Positive	Negative	Negative	Negative
#10	Positive	Negative	Negative	Negative
#11	Positive	Negative	Negative	Positive

TABLE S2. Detailed ASSURE test band profiles (NS1, IgM, IgG) for samples with discrepant IgG results when compared to the SD Bioline Dengue IgG test line (Bangladesh study).

Number	SD Bioline Dengue IgG	ASSURE Dengue Ab/Ag Rapid Test		
		NS1	IgM	IgG
#1	Positive	Positive	Negative	Negative
#2	Positive	Positive	Negative	Negative
#3	Positive	Positive	Positive	Negative
#4	Positive	Positive	Negative	Negative
#5	Positive	Negative	Negative	Negative
#6	Positive	Negative	Negative	Negative
#7	Positive	Positive	Positive	Negative
#8	Positive	Positive	Negative	Negative
#9	Positive	Negative	Negative	Negative
#10	Positive	Negative	Negative	Negative
#11	Positive	Negative	Negative	Negative
#12	Positive	Negative	Negative	Negative
#13	Positive	Negative	Negative	Negative
#14	Positive	Negative	Negative	Negative
#15	Positive	Negative	Negative	Negative
#16	Positive	Negative	Negative	Negative
#17	Positive	Negative	Negative	Negative
#18	Positive	Negative	Negative	Negative
#19	Positive	Negative	Negative	Negative
#20	Positive	Positive	Positive	Negative
#21	Positive	Positive	Positive	Negative
#22	Positive	Positive	Positive	Negative
#23	Positive	Negative	Positive	Negative

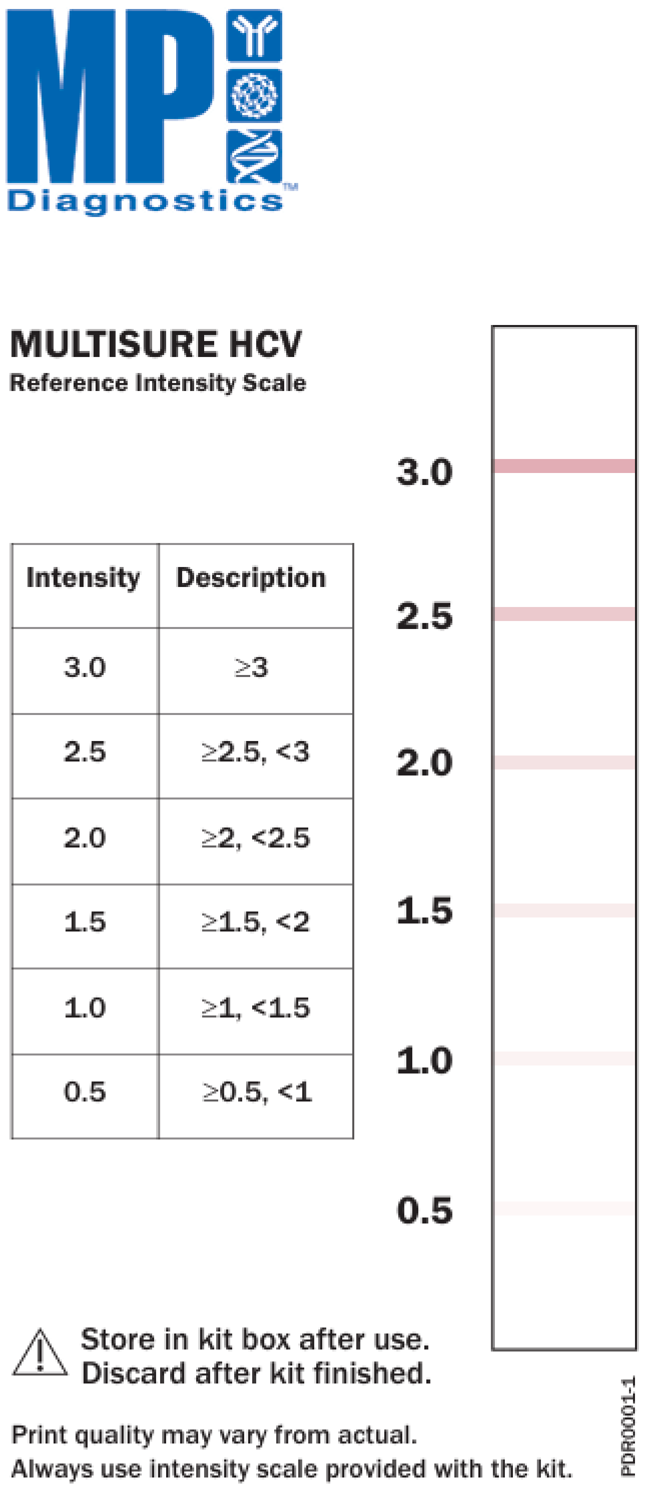

MP Diagnostics

MULTISURE HCV

Reference Intensity Scale

Intensity	Description
3.0	≥3
2.5	≥2.5, <3
2.0	≥2, <2.5
1.5	≥1.5, <2
1.0	≥1, <1.5
0.5	≥0.5, <1

Store in kit box after use.
Discard after kit finished.

Print quality may vary from actual.
Always use intensity scale provided with the kit.

PDR0001-1

FIGURE S1. Intensity Scale used for manual scoring in the specimen equivalency study

TABLE S3. Test line intensity scores for matched venous whole blood, finger-prick whole blood, plasma, and serum from 21 dengue-negative healthy donors in the specimen equivalency study.

Donor	Specimen	Replicate	Results				
			Control	IgG	IgM	NS1	Final Interpretation
1	Venous whole blood	1	0	0	0	0	Dengue Negative
		2	0	0	0	0	Dengue Negative
	Plasma	1	0	0	0	0	Dengue Negative
		2	0	0	0	0	Dengue Negative
	Serum	1	0	0	0	0	Dengue Negative
		2	0	0	0	0	Dengue Negative
	Finger prick whole blood	1	0	0	0	0	Dengue Negative
2	Venous whole blood	1	0	0	0	0	Dengue Negative
		2	0	0	0	0	Dengue Negative
	Plasma	1	0	0	0	0	Dengue Negative
		2	0	0	0	0	Dengue Negative
	Serum	1	0	0	0	0	Dengue Negative
		2	0	0	0	0	Dengue Negative
	Finger prick whole blood	1	0	0	0	0	Dengue Negative
3	Venous whole blood	1	0	0	0	0	Dengue Negative
		2	0	0	0	0	Dengue Negative
	Plasma	1	0	0	0	0	Dengue Negative
		2	0	0	0	0	Dengue Negative
	Serum	1	0	0	0	0	Dengue Negative
		2	0	0	0	0	Dengue Negative
	Finger prick whole blood	1	0	0	0	0	Dengue Negative
4	Venous whole blood	1	0	0	0	0	Dengue Negative
		2	0	0	0	0	Dengue Negative
	Plasma	1	0	0	0	0	Dengue Negative
		2	0	0	0	0	Dengue Negative
	Serum	1	0	0	0	0	Dengue Negative
		2	0	0	0	0	Dengue Negative
	Finger prick whole blood	1	0	0	0	0	Dengue Negative
5	Venous whole blood	1	0	0	0	0	Dengue Negative
		2	0	0	0	0	Dengue Negative
	Plasma	1	0	0	0	0	Dengue Negative
		2	0	0	0	0	Dengue Negative
	Serum	1	0	0	0	0	Dengue Negative
		2	0	0	0	0	Dengue Negative
	Finger prick whole blood	1	0	0	0	0	Dengue Negative
6	Venous whole blood	1	0	0	0	0	Dengue Negative
		2	0	0	0	0	Dengue Negative

Donor	Specimen	Replicate	Results				
			Control	IgG	IgM	NS1	Final Interpretation
	Plasma	1	0	0	0	0	Dengue Negative
		2	0	0	0	0	Dengue Negative
	Serum	1	0	0	0	0	Dengue Negative
		2	0	0	0	0	Dengue Negative
	Finger prick whole blood	1	0	0	0	0	Dengue Negative
7	Venous whole blood	1	0	0	0	0	Dengue Negative
		2	0	0	0	0	Dengue Negative
	Plasma	1	0	0	0	0	Dengue Negative
		2	0	0	0	0	Dengue Negative
	Serum	1	0	0	0	0	Dengue Negative
		2	0	0	0	0	Dengue Negative
	Finger prick whole blood	1	0	0	0	0	Dengue Negative
8	Venous whole blood	1	0	0	0	0	Dengue Negative
		2	0	0	0	0	Dengue Negative
	Plasma	1	0	0	0	0	Dengue Negative
		2	0	0	0	0	Dengue Negative
	Serum	1	0	0	0	0	Dengue Negative
		2	0	0	0	0	Dengue Negative
	Finger prick whole blood	1	0	0	0	0	Dengue Negative
9	Venous whole blood	1	0	0	0	0	Dengue Negative
		2	0	0	0	0	Dengue Negative
	Plasma	1	0	0	0	0	Dengue Negative
		2	0	0	0	0	Dengue Negative
	Serum	1	0	0	0	0	Dengue Negative
		2	0	0	0	0	Dengue Negative
	Finger prick whole blood	1	0	0	0	0	Dengue Negative
10	Venous whole blood	1	0	0	0	0	Dengue Negative
		2	0	0	0	0	Dengue Negative
	Plasma	1	0	0	0	0	Dengue Negative
		2	0	0	0	0	Dengue Negative
	Serum	1	0	0	0	0	Dengue Negative
		2	0	0	0	0	Dengue Negative
	Finger prick whole blood	1	0	0	0	0	Dengue Negative
11	Venous whole blood	1	0	0	0	0	Dengue Negative
		2	0	0	0	0	Dengue Negative
	Plasma	1	0	0	0	0	Dengue Negative
		2	0	0	0	0	Dengue Negative
	Serum	1	0	0	0	0	Dengue Negative
		2	0	0	0	0	Dengue Negative
	Finger prick whole blood	1	0	0	0	0	Dengue Negative

Donor	Specimen	Replicate	Results				
			Control	IgG	IgM	NS1	Final Interpretation
12	Venous whole blood	1	0	0	0	0	Dengue Negative
		2	0	0	0	0	Dengue Negative
	Plasma	1	0	0	0	0	Dengue Negative
		2	0	0	0	0	Dengue Negative
	Serum	1	0	0	0	0	Dengue Negative
		2	0	0	0	0	Dengue Negative
	Finger prick whole blood	1	0	0	0	0	Dengue Negative
13	Venous whole blood	1	0	0	0	0	Dengue Negative
		2	0	0	0	0	Dengue Negative
	Plasma	1	0	0	0	0	Dengue Negative
		2	0	0	0	0	Dengue Negative
	Serum	1	0	0	0	0	Dengue Negative
		2	0	0	0	0	Dengue Negative
	Finger prick whole blood	1	0	0	0	0	Dengue Negative
14	Venous whole blood	1	0	0	0	0	Dengue Negative
		2	0	0	0	0	Dengue Negative
	Plasma	1	0	0	0	0	Dengue Negative
		2	0	0	0	0	Dengue Negative
	Serum	1	0	0	0	0	Dengue Negative
		2	0	0	0	0	Dengue Negative
	Finger prick whole blood	1	0	0	0	0	Dengue Negative
15	Venous whole blood	1	0	0	0	0	Dengue Negative
		2	0	0	0	0	Dengue Negative
	Plasma	1	0	0	0	0	Dengue Negative
		2	0	0	0	0	Dengue Negative
	Serum	1	0	0	0	0	Dengue Negative
		2	0	0	0	0	Dengue Negative
	Finger prick whole blood	1	0	0	0	0	Dengue Negative
16	Venous whole blood	1	0	0	0	0	Dengue Negative
		2	0	0	0	0	Dengue Negative
	Plasma	1	0	0	0	0	Dengue Negative
		2	0	0	0	0	Dengue Negative
	Serum	1	0	0	0	0	Dengue Negative
		2	0	0	0	0	Dengue Negative
	Finger prick whole blood	1	0	0	0	0	Dengue Negative
17	Venous whole blood	1	0	0	0	0	Dengue Negative
		2	0	0	0	0	Dengue Negative
	Plasma	1	0	0	0	0	Dengue Negative
		2	0	0	0	0	Dengue Negative
	Serum	1	0	0	0	0	Dengue Negative

Donor	Specimen	Replicate	Results				
			Control	IgG	IgM	NS1	Final Interpretation
		2	0	0	0	0	Dengue Negative
	Finger prick whole blood	1	0	0	0	0	Dengue Negative
18	Venous whole blood	1	0	0	0	0	Dengue Negative
		2	0	0	0	0	Dengue Negative
	Plasma	1	0	0	0	0	Dengue Negative
		2	0	0	0	0	Dengue Negative
	Serum	1	0	0	0	0	Dengue Negative
		2	0	0	0	0	Dengue Negative
	Finger prick whole blood	1	0	0	0	0	Dengue Negative
19	Venous whole blood	1	0	0	0	0	Dengue Negative
		2	0	0	0	0	Dengue Negative
	Plasma	1	0	0	0	0	Dengue Negative
		2	0	0	0	0	Dengue Negative
	Serum	1	0	0	0	0	Dengue Negative
		2	0	0	0	0	Dengue Negative
	Finger prick whole blood	1	0	0	0	0	Dengue Negative
20	Venous whole blood	1	0	0	0	0	Dengue Negative
		2	0	0	0	0	Dengue Negative
	Plasma	1	0	0	0	0	Dengue Negative
		2	0	0	0	0	Dengue Negative
	Serum	1	0	0	0	0	Dengue Negative
		2	0	0	0	0	Dengue Negative
	Finger prick whole blood	1	0	0	0	0	Dengue Negative
21	Venous whole blood	1	0	0	0	0	Dengue Negative
		2	0	0	0	0	Dengue Negative
	Plasma	1	0	0	0	0	Dengue Negative
		2	0	0	0	0	Dengue Negative
	Serum	1	0	0	0	0	Dengue Negative
		2	0	0	0	0	Dengue Negative
	Finger prick whole blood	1	0	0	0	0	Dengue Negative

TABLE S4. Test line intensity scores for matched specimen types spiked with dengue IgG, IgM, or NS1 positive materials in the specimen equivalency study.

Donor	Specimen	Replicate	Results				
			Control	IgG	IgM	NS1	Final Interpretation
1	Venous whole blood	1	3	1.5	0	0	Dengue IgG Positive
		2	3	1.5	0	0	Dengue IgG Positive
	Plasma	1	3	1.5	0	0	Dengue IgG Positive
		2	3	1.5	0	0	Dengue IgG Positive
	Serum	1	3	1	0	0	Dengue IgG Positive
		2	3	1	0	0	Dengue IgG Positive
	Finger prick whole blood	1	3	1	0	0	Dengue IgG Positive
2	Venous whole blood	1	3	1.5	0	0	Dengue IgG Positive
		2	3	1	0	0	Dengue IgG Positive
	Plasma	1	3	1	0	0	Dengue IgG Positive
		2	3	1	0	0	Dengue IgG Positive
	Serum	1	3	1	0	0	Dengue IgG Positive
		2	3	1	0	0	Dengue IgG Positive
	Finger prick whole blood	1	3	1	0	0	Dengue IgG Positive
3	Venous whole blood	1	3	1.5	0	0	Dengue IgG Positive
		2	3	1.5	0	0	Dengue IgG Positive
	Plasma	1	3	1	0	0	Dengue IgG Positive
		2	3	1	0	0	Dengue IgG Positive
	Serum	1	3	1	0	0	Dengue IgG Positive
		2	3	1	0	0	Dengue IgG Positive
	Finger prick whole blood	1	3	1	0	0	Dengue IgG Positive
4	Venous whole blood	1	3	1	0	0	Dengue IgG Positive
		2	3	1	0	0	Dengue IgG Positive
	Plasma	1	3	1.5	0	0	Dengue IgG Positive
		2	3	1.5	0	0	Dengue IgG Positive
	Serum	1	3	1	0	0	Dengue IgG Positive
		2	3	1	0	0	Dengue IgG Positive
	Finger prick whole blood	1	3	1	0	0	Dengue IgG Positive
5	Venous whole blood	1	3	1.5	0	0	Dengue IgG Positive
		2	3	1.5	0	0	Dengue IgG Positive
	Plasma	1	3	1.5	0	0	Dengue IgG Positive
		2	3	1.5	0	0	Dengue IgG Positive
	Serum	1	3	1	0	0	Dengue IgG Positive
		2	3	1	0	0	Dengue IgG Positive
	Finger prick whole blood	1	3	1	0	0	Dengue IgG Positive
6	Venous whole blood	1	3	1.5	0	0	Dengue IgG Positive
		2	3	1.5	0	0	Dengue IgG Positive
	Plasma	1	3	1	0	0	Dengue IgG Positive
		2	3	1	0	0	Dengue IgG Positive

Donor	Specimen	Replicate	Results				
			Control	IgG	IgM	NS1	Final Interpretation
	Serum	1	3	1	0	0	Dengue IgG Positive
		2	3	1	0	0	Dengue IgG Positive
	Finger prick whole blood	1	3	1	0	0	Dengue IgG Positive
7	Venous whole blood	1	3	1.5	0	0	Dengue IgG Positive
		2	3	1.5	0	0	Dengue IgG Positive
	Plasma	1	3	1	0	0	Dengue IgG Positive
		2	3	1	0	0	Dengue IgG Positive
	Serum	1	3	1.5	0	0	Dengue IgG Positive
		2	3	1.5	0	0	Dengue IgG Positive
	Finger prick whole blood	1	3	1	0	0	Dengue IgG Positive
8	Venous whole blood	2	3	0	1.5	0	Dengue IgM Positive
		2	3	0	1.5	0	Dengue IgM Positive
	Plasma	1	3	0	1.5	0	Dengue IgM Positive
		2	3	0	1.5	0	Dengue IgM Positive
	Serum	1	3	0	1	0	Dengue IgM Positive
		2	3	0	1	0	Dengue IgM Positive
	Finger prick whole blood	1	3	0	1.5	0	Dengue IgM Positive
9	Venous whole blood	1	3	0	1	0	Dengue IgM Positive
		2	3	0	1	0	Dengue IgM Positive
	Plasma	1	3	0	1	0	Dengue IgM Positive
		2	3	0	1	0	Dengue IgM Positive
	Serum	1	3	0	1	0	Dengue IgM Positive
		2	3	0	1	0	Dengue IgM Positive
	Finger prick whole blood	1	3	0	1	0	Dengue IgM Positive
10	Venous whole blood	1	3	0	1	0	Dengue IgM Positive
		2	3	0	1	0	Dengue IgM Positive
	Plasma	1	3	0	1	0	Dengue IgM Positive
		2	3	0	1	0	Dengue IgM Positive
	Serum	1	3	0	1	0	Dengue IgM Positive
		2	3	0	1	0	Dengue IgM Positive
	Finger prick whole blood	1	3	0	1	0	Dengue IgM Positive
11	Venous whole blood	1	3	0	1.5	0	Dengue IgM Positive
		2	3	0	1.5	0	Dengue IgM Positive
	Plasma	1	3	0	1	0	Dengue IgM Positive
		2	3	0	1	0	Dengue IgM Positive
	Serum	1	3	0	1	0	Dengue IgM Positive
		2	3	0	1	0	Dengue IgM Positive
	Finger prick whole blood	1	3	0	1	0	Dengue IgM Positive
12	Venous whole blood	1	3	0	1.5	0	Dengue IgM Positive
		2	3	0	1.5	0	Dengue IgM Positive

Donor	Specimen	Replicate	Results				
			Control	IgG	IgM	NS1	Final Interpretation
	Plasma	1	3	0	1.5	0	Dengue IgM Positive
		2	3	0	1.5	0	Dengue IgM Positive
	Serum	1	3	0	1	0	Dengue IgM Positive
		2	3	0	1	0	Dengue IgM Positive
	Finger prick whole blood	1	3	0	1	0	Dengue IgM Positive
13	Venous whole blood	1	3	0	1	0	Dengue IgM Positive
		2	3	0	1	0	Dengue IgM Positive
	Plasma	1	3	0	1.5	0	Dengue IgM Positive
		2	3	0	1.5	0	Dengue IgM Positive
	Serum	1	3	0	1.5	0	Dengue IgM Positive
		2	3	0	1.5	0	Dengue IgM Positive
	Finger prick whole blood	1	3	0	1	0	Dengue IgM Positive
14	Venous whole blood	1	3	0	1.5	0	Dengue IgM Positive
		2	3	0	1.5	0	Dengue IgM Positive
	Plasma	1	3	0	1.5	0	Dengue IgM Positive
		2	3	0	1.5	0	Dengue IgM Positive
	Serum	1	3	0	1	0	Dengue IgM Positive
		2	3	0	1	0	Dengue IgM Positive
	Finger prick whole blood	1	3	0	1	0	Dengue IgM Positive
15	Venous whole blood	1	3	0	0	1.5	Dengue NS1 Positive
		2	3	0	0	1.5	Dengue NS1 Positive
	Plasma	1	3	0	0	1.5	Dengue NS1 Positive
		2	3	0	0	1.5	Dengue NS1 Positive
	Serum	1	3	0	0	1.5	Dengue NS1 Positive
		2	3	0	0	1.5	Dengue NS1 Positive
	Finger prick whole blood	1	3	0	0	1.5	Dengue NS1 Positive
16	Venous whole blood	1	3	0	0	1.5	Dengue NS1 Positive
		2	3	0	0	1.5	Dengue NS1 Positive
	Plasma	1	3	0	0	1.5	Dengue NS1 Positive
		2	3	0	0	1.5	Dengue NS1 Positive
	Serum	1	3	0	0	1.5	Dengue NS1 Positive
		2	3	0	0	1.5	Dengue NS1 Positive
	Finger prick whole blood	1	3	0	0	1.5	Dengue NS1 Positive
17	Venous whole blood	1	3	0	0	1.5	Dengue NS1 Positive
		2	3	0	0	1.5	Dengue NS1 Positive
	Plasma	1	3	0	0	1.5	Dengue NS1 Positive
		2	3	0	0	1.5	Dengue NS1 Positive
	Serum	1	3	0	0	1.5	Dengue NS1 Positive
		2	3	0	0	1.5	Dengue NS1 Positive
	Finger prick whole blood	1	3	0	0	1.5	Dengue NS1 Positive

Donor	Specimen	Replicate	Results				
			Control	IgG	IgM	NS1	Final Interpretation
18	Venous whole blood	1	3	0	0	1.5	Dengue NS1 Positive
		2	3	0	0	1.5	Dengue NS1 Positive
	Plasma	1	3	0	0	1.5	Dengue NS1 Positive
		2	3	0	0	1.5	Dengue NS1 Positive
	Serum	1	3	0	0	1.5	Dengue NS1 Positive
		2	3	0	0	1.5	Dengue NS1 Positive
	Finger prick whole blood	1	3	0	0	1.5	Dengue NS1 Positive
19	Venous whole blood	1	3	0	0	1.5	Dengue NS1 Positive
		2	3	0	0	1.5	Dengue NS1 Positive
	Plasma	1	3	0	0	1.5	Dengue NS1 Positive
		2	3	0	0	1.5	Dengue NS1 Positive
	Serum	1	3	0	0	1.5	Dengue NS1 Positive
		2	3	0	0	1.5	Dengue NS1 Positive
	Finger prick whole blood	1	3	0	0	1	Dengue NS1 Positive
20	Venous whole blood	1	3	0	0	1.5	Dengue NS1 Positive
		2	3	0	0	1.5	Dengue NS1 Positive
	Plasma	1	3	0	0	1.5	Dengue NS1 Positive
		2	3	0	0	1.5	Dengue NS1 Positive
	Serum	1	3	0	0	1.5	Dengue NS1 Positive
		2	3	0	0	1.5	Dengue NS1 Positive
	Finger prick whole blood	1	3	0	0	1.5	Dengue NS1 Positive
21	Venous whole blood	1	3	0	0	1.5	Dengue NS1 Positive
		2	3	0	0	1.5	Dengue NS1 Positive
	Plasma	1	3	0	0	1.5	Dengue NS1 Positive
		2	3	0	0	1.5	Dengue NS1 Positive
	Serum	1	3	0	0	1.5	Dengue NS1 Positive
		2	3	0	0	1.5	Dengue NS1 Positive
	Finger prick whole blood	1	3	0	0	1	Dengue NS1 Positive
