# Supplementary material for: Multicenter evaluation of a single-device dengue antibody/antigen rapid test for simultaneous detection of NS1, IgM, and IgG
Source: Microbiol Spectr. 2026 May 18;14(7):e03669-25. doi: 10.1128/spectrum.03669-25 (PMC13339962; doi:10.1128/spectrum.03669-25)
Supplement: Reviewer comments [file reviewer-comments.pdf]

AMERICAN SOCIETY FOR MICROBIOLOGY | Microbiology Spectrum

Multicenter Evaluation of a Single-Device Dengue Antibody/Antigen Rapid Test for Simultaneous Detection of NS1, IgM, and IgG

Zhihai ZHAO, Mettingal Shincy, Lay Teoh, KL Ravikumar, Haris Ong, Matthew Mak, Yang Du, Mohammad Shafiul Alam, and Na Xu

Corresponding Author(s): Zhihai ZHAO, MP Biomedicals LLC

Review Timeline:

Submission Date:	January 8, 2026
Editorial Decision:	April 4, 2026
Revision Received:	April 8, 2026
Accepted:	April 10, 2026

Editor: Day-Yu Chao

Reviewer(s): The reviewers have opted to remain anonymous.

Transaction Report:

(Note: With the exception of the correction of typographical or spelling errors that could be a source of ambiguity, letters and reports are not edited. The original formatting of letters and referee reports may not be reflected in this compilation.)

DOI: https://doi.org/10.1128/spectrum.03669-25

Re: Spectrum03669-25 (Multicenter Evaluation of a Single-Device Dengue Antibody/Antigen Rapid Test for Simultaneous Detection of NS1, IgM, and IgG)

Dear Dr. Zhihai ZHAO:

Thank you for the privilege of reviewing your work. Below you will find my comments, instructions from the Spectrum editorial office, and the reviewer comments.

The authors have substantially revised the manuscript to reach the publication level, except for one thing: the use of FFU as the detection limit of NS1 antigens. It should be noted that the amount of NS1 antigen secreted into the tissue culture supernatant during virus infection is not quantifiable by FFU and may vary depending on the virus strain used. One focus or FFU refers to a single virus infection of the cells, and it definitely does not refer to the amount of NS1 antigen. The only way to accurately quantify the NS1 antigen in the culture supernatant is to purify it. There are many commercial sources from which the authors could obtain purified NS1 derived from four DENV serotypes. This should be discussed in the manuscript.

Please return the manuscript within 60 days; if you cannot complete the modification within this time period, please contact me. If you do not wish to modify the manuscript and prefer to submit it to another journal, notify me immediately so that the manuscript may be formally withdrawn from consideration by Spectrum.

Revision Guidelines
To submit your modified manuscript, log into the submission site at https://spectrum.msubmit.net/cgi-bin/main.plex. Go to Author Tasks and click the appropriate manuscript title to begin. The information you entered when you first submitted the paper will be displayed; update this as necessary. Note the following requirements:

• Upload point-by-point responses to the issues raised by the reviewers in a file named "Response to Reviewers," NOT in your cover letter.
• Upload a compare copy of the manuscript (without figures) as a "Marked-Up Manuscript" file.
• Upload a clean .DOC/.DOCX version of the revised manuscript and remove the previous version.
• Each figure must be uploaded as a separate, editable, high-resolution file (TIFF or EPS preferred), and any multipanel figures must be assembled into one file.
• Any supplemental material intended for posting by ASM should be uploaded with their legends separate from the main manuscript. You can combine all supplemental material into one file (preferred) or split it into a maximum of 10 files with all associated legends included.

For complete guidelines on revision requirements, see our Submission and Review Process webpage. Submission of a paper that does not conform to guidelines may delay acceptance of your manuscript.

Data availability: ASM policy requires that data be available to the public upon online posting of the article, so please verify all links to sequence records, if present, and make sure that each number retrieves the full record of the data. If a new accession number is not linked or a link is broken, provide Spectrum production staff with the correct URL for the record. If the accession numbers for new data are not publicly accessible before the expected online posting of the article, publication may be delayed; please contact production staff (Spectrum@asmusa.org) immediately with the expected release date.

Publication Fees: For information on publication fees and which article types are subject to charges, visit our website. If your manuscript is accepted for publication and any fees apply, you will be contacted separately about payment during the production process; please follow the instructions in that e-mail. Arrangements for payment must be made before your article is published.

ASM Membership: Corresponding authors may join or renew ASM membership to obtain discounts on publication fees. Need to upgrade your membership level? Please contact Customer Service at Service@asmusa.org.

The ASM Journals program strives for constant improvement in our submission and publication process. Please tell us how we can improve your experience by taking this quick Author Survey.

Thank you for submitting your paper to Spectrum.

Sincerely,
Day-Yu Chao
Editor
Microbiology Spectrum

Reviewer #2 (Comments for the Author):

Zhao et al. conducted a multicenter study evaluating a single-device rapid test for the simultaneous detection of NS1, IgM, and IgG. The study aimed to differentiate between primary and secondary dengue infections and compared diagnostic performance against reference tests, such as ELISA. This device shows significant potential for use in rapid point-of-care testing.
This is a revised manuscript, and the authors have addressed all previous reviewer concerns. The updated manuscript is significantly improved and falls well within the scope of this journal.

The authors have substantially revised the manuscript to reach the publication level, except for one thing: the use of FFU as the detection limit of NS1 antigens. It should be noted that the amount of NS1 antigen secreted into the tissue culture supernatant during virus infection is not quantifiable by FFU and may vary depending on the virus strain used. One focus or FFU refers to a single virus infection of the cells, and it definitely does not refer to the amount of NS1 antigen. The only way to accurately quantify the NS1 antigen in the culture supernatant is to purify it. There are many commercial sources from which the authors could obtain purified NS1 derived from four DENV serotypes. This should be discussed in the manuscript.

Response: We thank the editor for this important and constructive comment. We fully agree that focus-forming units per milliliter (ffu/mL) cannot be used as a direct quantitative measure of NS1 antigen levels in tissue culture supernatants, and we appreciate the opportunity to address this limitation explicitly in the manuscript.

In response, we have added the following discussion to the manuscript (page [20], lines [383-392]):

"While the analytical sensitivity studies using live dengue virus cultures provided valuable comparative data across all four DENV serotypes, it is important to acknowledge a key limitation in the use of focus-forming units per milliliter (ffu/mL) as a proxy for NS1 antigen levels. The ffu quantifies infectious viral particles capable of forming foci in cell culture, but the amount of NS1 antigen secreted during viral infection may vary significantly depending on the viral strain, cell line, and culture conditions used (27–30). A single focus-forming unit represents one infectious viral particle, not a standardized amount of NS1 antigen. However, the parallel testing of multiple commercial rapid diagnostic tests (SD Bioline Dengue Duo and SD Biosensor Standard Q Dengue Duo) using the same viral culture samples validated NS1 presence and provided a basis for comparing relative antigen levels across different platforms under identical testing conditions."

We agree that purified NS1 antigens would provide more accurate quantification, and we acknowledge the availability of commercial sources for purified NS1 from all four DENV serotypes. However, our study design aimed to evaluate real-world performance using authentic viral culture conditions that more closely simulate clinical samples. Moreover, the parallel testing with established commercial RDTs (SD Bioline Dengue Duo and SD Biosensor Standard Q Dengue Duo) using identical culture samples served as an additional validation step and provided a comparative framework for assessing relative NS1 detection capabilities across different platforms under standardized conditions.

We believe the added discussion adequately addresses the editor's concern while providing appropriate context for interpreting our analytical sensitivity results. The limitation is now clearly stated, and readers can better interpret the FFU-based data within this framework.

Re: Spectrum03669-25R1 (Multicenter Evaluation of a Single-Device Dengue Antibody/Antigen Rapid Test for Simultaneous Detection of NS1, IgM, and IgG)

Dear Dr. Zhihai ZHAO:

Your manuscript has been accepted, and I am forwarding it to the ASM production staff for publication. Your paper will first be checked to make sure all elements meet the technical requirements. ASM staff will contact you if anything needs to be revised before copyediting and production can begin. Otherwise, you will be notified when your proofs are ready to be viewed.

Data Availability: ASM policy requires that data be available to the public upon online posting of the article, so please verify all links to sequence records, if present, and make sure that each number retrieves the full record of the data. If a new accession number is not linked or a link is broken, provide production staff with the correct URL for the record. If the accession numbers for new data are not publicly accessible before the expected online posting of the article, publication may be delayed; please contact ASM production staff immediately with the expected release date.

Publication Fees: For information on publication fees and which article types have charges, please visit our website. We have partnered with Copyright Clearance Center (CCC) to collect author charges. If fees apply to your paper, you will receive a message from no-reply@copyright.com with further instructions. For questions related to paying charges through RightsLink, please contact CCC at ASM_Support@copyright.com or toll free at +1-877-622-5543. CCC makes every attempt to respond to all emails within 24 hours.

ASM Membership: Corresponding authors may join or renew ASM membership to obtain discounts on publication fees. Need to upgrade your membership level? Please contact Customer Service at Service@asmusa.org.

Embargo Policy: A press release may be issued as soon as the manuscript is posted on the Spectrum Latest Articles webpage. The corresponding author will receive an email with the subject line "ASM Journals Author Services Notification" when the article is available online.

The ASM Journals program strives for constant improvement in our submission and publication process. Please tell us how we can improve your experience by taking this quick Author Survey.

Thank you for submitting your paper to Spectrum.

Sincerely,
Day-Yu Chao
Editor
Microbiology Spectrum
